# Lipid Profile and Serum Characteristics of the Blind Subterranean Mole Rat, *Spalax*

**DOI:** 10.1371/journal.pone.0004528

**Published:** 2009-02-20

**Authors:** Nicola J. Nasser, Marielle Kaplan, Eviatar Nevo, Michael Aviram

**Affiliations:** 1 Department of Oncology, Shaare Zedek Medical Center, Jerusalem, Israel; 2 Clinical Biochemistry Laboratory, Rambam Medical Center, Haifa, Israel; 3 Institute of Evolution, International Graduate Center of Evolution, University of Haifa, Haifa, Israel; 4 Lipid Research Laboratory, Technion Faculty of Medicine, The Rappaport Family Institute for Research in the Medical Sciences, Rambam Medical Center, Haifa, Israel; Universidad Europea de Madrid, Spain

## Abstract

**Background:**

*Spalax* (blind subterranean mole rat), is a mammal adapted to live in fluctuating oxygen levels, and can survive severe hypoxia and hypercapnia. The adaptive evolution of *Spalax* to underground life resulted in structural and molecular-genetic differences comparing to above-ground mammals. These differences include higher myocardial maximal oxygen consumption, increased lung diffusion capacity, increased blood vessels density, and unique expression patterns of cancer and angiogenesis related genes such as heparanase, vascular endothelial growth factor, and P53.

**Methodology/Principal Findings:**

Here we elucidate the main characteristics of *Spalax* lipid profile, as well as its main antioxidant and serum parameters. Compared to human, *Spalax* possesses lower total-cholesterol, low density lipoproteins (LDL) and triglycerides levels, and higher levels of high density lipoproteins (HDL). Apolipoprotein A-I and apolipoprotein B-100 were significantly lower in *Spalax* compared to human. Paraoxonase (PON) 1 arylesterase activity, was higher in *Spalax* compared to both human and mouse serum levels. Analysis of serum chemistry of *Spalax* revealed special features in this mammal.

**Conclusions/Significance:**

*Spalax* possesses a unique lipid profile with high HDL and low LDL lipoproteins. The antioxidant serum content in the mole rat is higher than that of human and mouse. Serum C reactive protein (CRP) levels are significantly lower in *Spalax* compared to that of human or mouse, reflecting low levels of inflammation. These differences between *Spalax*, human and mouse are due to several factors including the intensive activity life-style that *Spalax* pursue underground, dietary components, and evolutionary genetic adaptations. Unfolding the genetic basis of these differences will probably result in unique treatments for a variety of human diseases such as dyslipedemias, inflammation and cancer.

## Introduction

The subterranean blind mole rat of the genus *Spalax* in Israel belongs to the mammalian superspecies *Spalax ehrenbergi* and the family *Spalacidae* [1], [2]. *Spalax* lives all its life, averaging three years, in sealed underground tunnels [2]. The oxygen and carbon dioxide levels inside the underground *Spalax* habitat fluctuate from values similar to those detected above ground to severe hypoxia and hypercapnia [3]. In laboratory conditions, *Spalax* survives oxygen levels as low as 2–3% for several hours, in contrast to above ground rats that die after less than 3 hours of exposure to such circumstances [4]. Several mechanisms account for the increased tolerance of *Spalax* to hypoxia. These mechanisms include higher myocardial maximal oxygen consumption [5], decreased diffusion distance of oxygen to the mitochondria [6], increase in the lung diffusion capacity [6], and specific differences in myoglobin which augment oxygen delivery at low oxygen tensions [7]. Blood vessels density in *Spalax* muscles is significantly higher than that of rat [8]–[10]. Vascular endothelial growth factor [9], [10], hypoxia inducible factor [11], and heparanase (an angiogenesis related enzyme) [12], [13] are constitutively high in some *Spalax* tissues [9]–[11]. Unique splice variants of heparanase were found in some *Spalax* tissues [12], [13]. *P53* gene in healthy *Spalax* individuals possesses amino acid substitutions in its DNA binding domain identical to mutations found in human tumors [14], [15]. These adaptive substitutions endow *Spalax* p53 with several-fold higher activation of cell arrest and DNA repair genes compared to human p53, and favor activation of DNA repair genes over apoptotic genes [14], [15]. Specific features of other molecules such as hemoglobin, haptoglobin, neuroglobin and cytoglobin were described in *Spalax* as well [1], [2].

*Spalax* daily life involves strenuous physical activity [1], [2]. *Spalax* digs to search for food (primarily subterranean plant organs such as roots, corms, bulbs), maintain its underground constructions, and search for mating partners [1], [2]. This probably explains the highly developed muscle mass that this mammal possesses. All the above characteristics of *Spalax* led us to investigate its lipid profile, serum oxidation parameters, and serum chemistry.

## Results

### *Spalax* lipid profile

**Figure 1:**
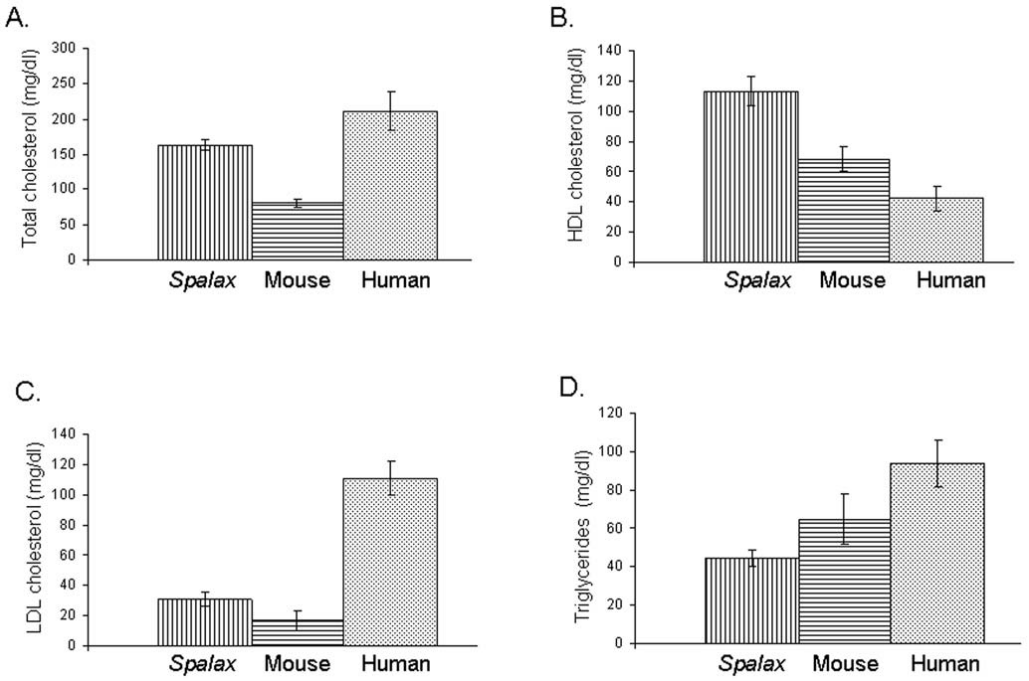
Comparison of mole rat (*Spalax*), mouse and human lipid profiles. A. Total cholesterol levels (mg/dl). B. High Density Lipoprotein cholesterol (HDL) levels (mg/dl). C. Low Density Lipoprotein cholesterol (LDL) levels (mg/dl). D. Triglycerides levels (mg/dl). Each bar represents a mean of three measurements from different individuals. Error bars represents standard deviations.

**Figure 2:**
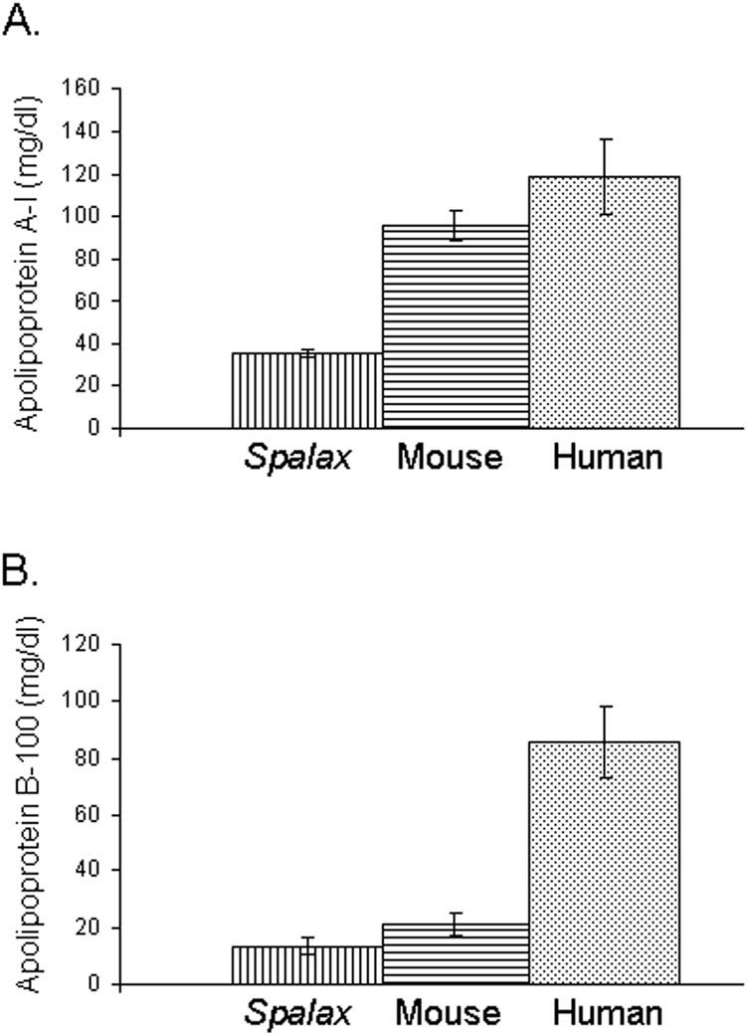
Comparison of mole rat (*Spalax*), mouse and human serum apolipoprotein A-I and apolipoprotein B-100. A. Apolipoprotein A-I was measured in mole rat (*Spalax*) (n = 3), mouse (n = 3), and human (n = 3). Apolipoprotein A-I of mole rat was 35±2 mg/dl, of mouse 96±7 mg/dl, and of human 119±17 mg/dl (mean±SD). B. Apolipoprotein B-100 of mole rat was 13±3 mg/dl, compared to 21±4 mg/dl of mouse, and 86±13 mg/dl of human. Bars represent mean values, and error bars represents standard deviations.

Lipid profile was measured in serum samples obtained from 3 mole rat (*Spalax judaei*) females, captured in the field in the day of the experiment. *Spalax* lipid profile was compared to those of mouse and human. Mole rat total cholesterol (mean±SD) was 164±8 mg/dl, compared to 80±5 mg/dl in mouse (p<0.0001) and 212±27 mg/dl in human (p<0.05) (Figure 1A). High density lipoprotein of mole rat was 113±10 mg/dl, versus 68±9 mg/dl in mouse (p<0.004) and 42±8 mg/dl in human (p<0.0006) (Figure 1B). Low density lipoprotein of mole rat was 31±5 mg/dl, compared to 17±7 mg/dl in mouse (p<0.04) and 111±11 mg/dl in human (p<0.0004) (Figure 1C). The average serum triglycerides level of mole rat was 44±4 mg/dl, compared to 65±13 mg/dl in mouse (p = 0.06) and 94±12 mg/dl in human (p<0.003) (Figure 1D). Apolipoprotein A-I and Apolipoprotein B-100 were also determined in serum of mole rat, mouse, and human. Mole rat Apolipoprotein A-I was 35±2 mg/dl, while its level in mouse was 96±7 mg/dl (p<0.0002), and in human 119±17 mg/dl (p<0.002) (Figure 2A). Apolipoprotein B-100 of mole rat was 13±3 mg/dl, compared to 21±4 mg/dl in mouse (p<0.053), and 86±13 mg/dl in human (p<0.0007) (Figure 2B).

### Mole rat (*Spalax*) serum oxidation

**Figure 3:**
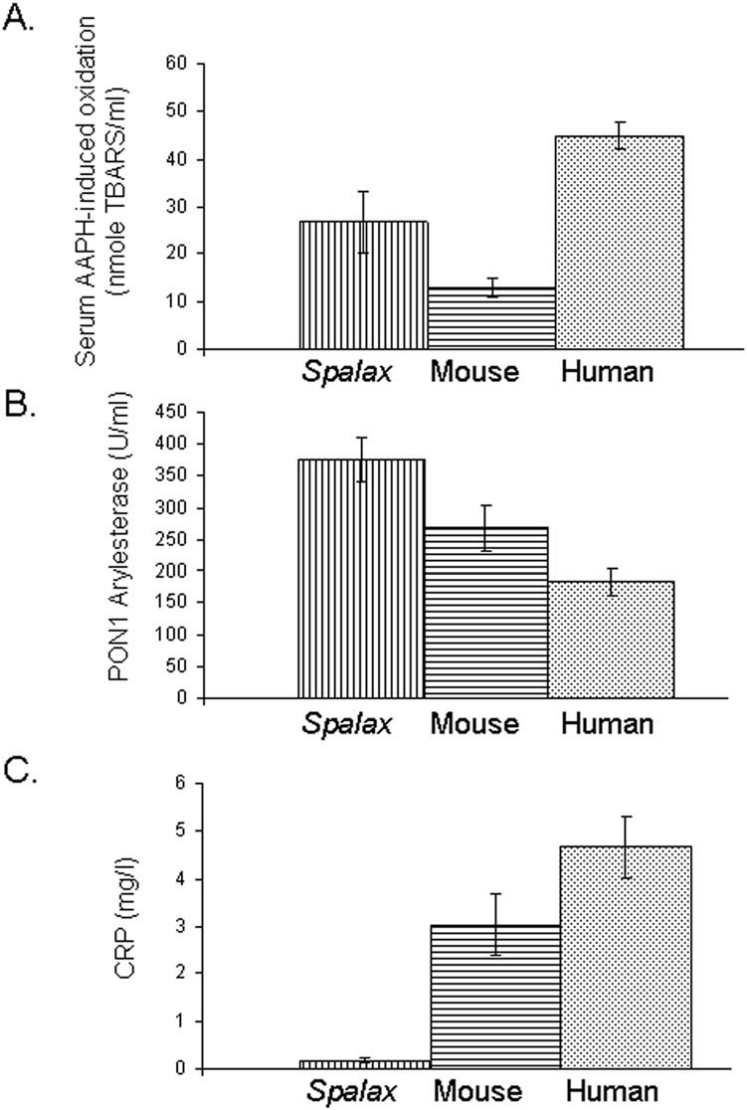
Comparison of mole rat (*Spalax*), mouse and human serum AAPH-induced oxidation and PON1 arylesterase. A. AAPH-induced oxidation of *Spalax* was 27±7 (nmole TBARS/ml) compared to 13±2 (nmole TBARS/ml) in mouse, and 45±3 (nmole TBARS/ml) in human. B. PON1 arylesterase of mole rat was 375±35 (U/ml), while its level in mouse was 269±36 (U/ml), and in human 182±21 (U/ml). C. C reactive protein (CRP) of mole rat was 0.18±0.06 (mg/L), of mouse 3.03±0.65 (mg/L), and of human 4.67±0.65 (mg/L). Bars represent mean values, and error bars represents standard deviations.

Serum AAPH-induced oxidation of *Spalax* was 27±7 (nmole TBARS/ml) compared to 13±2 (nmole TBARS/ml) in mouse (p<0.03), and 45±3 (nmole TBARS/ml) in human (p<0.02) (Figure 3A). Paraoxonase (PON) 1 arylesterase (an enzyme present on the HDL liporopotein that possesses antioxidants properties) level in mole rat serum was 375±35 (U/ml), while its level in mouse was 269±36 (U/ml) (p<0.03), and in human 182±21 (U/ml) (p<0.0015) (Figure 3B). C reactive protein (CRP), an acute phase protein produced by the liver and by adipocytes, was prominently lower in mole rat (0.18±0.06 (mg/L)) than in mouse (3.03±0.65 (mg/L)) and human (4.67±0.65 (mg/L)) (p values<0.002 and <0.0003 respectively) (Figure 3C).

### *Spalax* serum chemistry

*Spalax* serum chemistry was determined from three mole rat females, which appeared to be pregnant. Mole rat serum chemistry was compared to that of human (Table 1). In most parameters the differences between mole rat and human proved statistically significant. Of special interest are the higher magnesium, phosphorus, and amylase levels in *Spalax*. High glucose levels detected in mole rats may be due to the fact that the measurement was not performed after 12 hours fasting as in humans. High alkaline phosphatase levels of *Spalax* may be due to pregnancies that were detected in the study individuals.

**Table 1:** Comparison of *Spalax* (mole rat) and human serum chemistries.

	*Spalax*	Human	P value
	Mean±SD	Mean±SD	
BUN (mg/dl)	38±5	16±3	<0.003
Glucose (mg/dl)	155±31	85±6	<0.02
Calcium (mg/dl)	11±1	9.3±0.4	= 0.053
Creatinine (mg/dl)	0.27±0.06	0.84±0.05	<0.0003
Phosphors (mg/dl)	8.7±0.2	3.4±0.3	<0.0001
GGT (U/l)	11.66±2.51	33±4	<0.001
Magnesium (mg/dl)	6.00±0.2	1.7±0.2	<0.0001
ALP (U/l)	922±60	84±9	<0.0001
LDH (U/l)	236±26	146±8	<0.005
Amylase (U/l)	1442±102	129±19	<0.0001
Sodium (mmol/l)	152±3	137±2	<0.002
Chloride (mmol/l)	119±3	106±3	<0.006
Total Protein (g/dl)	8.1±0.6	7.1±0.3	<0.05
Albumin (g/dl)	2.6±0.2	4.6±0.3	<0.0006
Uric Acid (mg/L)	0.6±0.1	6.1±0.6	<0.0001

BUN = Blood Urea Nitrogen; GGT = Gamma-Glutamyl Transpeptidase; ALP = Alkaline Phosphatase; LDH = Lactate Dehydrogenase.

## Discussion

### *Spalax* adaptation to underground life

*Spalax* is a mammal that is adapted to tolerate severe hypoxia [1]–[11]. *Spalax* evolutionary ancestors went underground due to planetary climatic savanization some 40 million years ago [1]. This change in *Spalax* living habitat was associated with several advantages and disadvantages, which elicited evolutionary, structural and genetic adaptations [1], [2]. Among the advantages that *Spalax* gained from residing in underground tunnels was protection from above ground predators, a relatively constant temperature and humidity, and access to underground food stores (plant roots) without many competitors. The “price” that *Spalax* paid for it was high: it encountered darkness, hypoxia, hypercapnia, complex energetics, poor food, and high pathogenicity it had to adaptively combat. The underground tunnels' oxygen and carbon dioxide content fluctuate dramatically, especially in rainy weather, when water fills a large volume of the *Spalax* burrows [3]. *Spalax* structural adaptations to the underground environment resulted in regression in some organs and progression of others [1]. Among the organs that regressed during *Spalax* evolution: I) the eyes which turned to be atrophic and subcutaneous. Actually, *Spalax* is unable to see, but still can discriminate between light and darkness i.e. it exercises photoperiodic perception, and maintain a circadian rhythm cycle [16]; II) *Spalax* limbs have been reduced in order to adapt to the small tunnels' dimensions; and III) the tail which disappeared to probably allow easier movement [1], [2]. Other organs of *Spalax* progressed as part of the evolutionary process. These include the muscles, both heart and skeletal muscles, which hypertrophied in order to sustain the tremendous efforts that *Spalax* exerts; the teeth which turned to be big and strong allowing *Spalax* to burrow its way through underground rocks and heavy soil [1], [2]; and the brain of *Spalax* which is significantly larger than that of rats, probably assisting it in managing through the extremely stressful life underground [1], [2].

### Lipid profile of *Spalax*

*Spalax* lipid profile shows that this mammal has low total cholesterol and low density lipoprotein (LDL) on the one hand and high levels of high density lipoproteins (HDL) on the other hand (Figure 1). Similar results were found in mouse compared to human, but HDL levels of *Spalax* were higher than in mouse (Figure 1B). The explanation for this unique lipid profile of *Spalax* may be due to the strenuous activity that *Spalax* peruses in its daily life [1], [2]. This activity may demand degradation of lipids which is mirrored as high serum HDL. The high HDL level culminates in low LDL levels, as LDL is calculated by decreasing the amount of HDL from total cholesterol together with 20% of the triglyceride levels [17]. Other factors that may contribute to this special lipid profile may be the unique diet that *Spalax* consumes, which is composed mainly from plant roots and geophytes. Changes in diet of human [18], and animal models [19] were associated with changes in their lipid profiles; further studies are necessary to elucidate possible positive characteristics of the diet of this unique mammal. Genetic evolution of *Spalax* may be one of the key factors that shape this lipid profile. Differences of about 15% were found in a variety of *Spalax* genes compared to human and other rodents [11]–[13]. Moreover, unique splice variants were found in some genes of *Spalax* that were not described yet in other mammals [12], [13]. Cloning the genes related to lipid metabolism will elucidate possible special characteristics that may contribute to this lipid phenotype of *Spalax*. Sequencing and analysis of the *Spalax* genome will help to unravel the nature of the genetic adaption to hypoxia. Further studies focusing on lipid profiles of other subterranean mammals (e.g. naked mole rat [20], [21], [22], pocket gopher [23], and golden mole [24]) will allow broader comparison with *Spalax* lipid profile, and probably elucidate the evolutionary processes behind its features.

### *Spalax* serum oxidation

The mole rat serum exhibits features of a low oxidative stress status, compared to human and mouse. Moreover, the antioxidant serum content measured in the mole rat was higher than that of human and mouse. These results could be related to the adaptive evolution of this mammal to underground stresses, especially to hypoxia and darkness. Interestingly, high oxidative damage levels were reported in the longest-living rodent, the naked mole-rat [21], [22], as opposed to *Spalax*. The CRP serum level which reflects the inflammatory status of the animal is especially lower in *Spalax* compared to that of human and mouse. These results of *Spalax* serum come in accordance with previous study that found low oxidative status in *Spalax* harderean gland [25]. This very low baseline of oxidative stress and inflammatory burden could be of interest to study the early stages of development of atherosclerosis, which is triggered by both increasing oxidative stress and inflammation.

### *Spalax* serum chemistry

Our current study elucidates special characteristics of *Spalax* serum. Higher magnesium, phosphorus, glucose, and amylase levels were detected in *Spalax* compared to human. The higher serum glucose level in *Spalax* may be partly due to non-fasting measurements or due to the pregnancies found in part of the study individuals. Further studies focusing on measuring fasting glucose will elucidate this point. Serum amylase was very high in *Spalax* (Table 1). The source of this enzyme in *Spalax* may be pancreatic or salivary. Serum magnesium and phosphoros were also high in *Spalax* compared to human.

### *Spalax* as a model organism for the study of hypoxia and dyslipidemias

This study defines the *Spalax* lipid profile, and its serum oxidative and biochemical properties. These results show that *Spalax* serum profile is different not just from that of human, but from mouse as well. *Spalax* is an HDL animal with high HDL cholesterol and low LDL serum levels. Further studies focusing on the evolutionary mechanisms that led to these differences are undergoing. We hope that understanding the *Spalax* genetic adaptation to hypoxia will help us to utilize similar mechanisms in managing human diseases related to hypoxia and elucidate ways to decrease LDL and increase HDL cholesterol in human.

## Materials and Methods

### Animals

#### Mole rats

The animals used for *Spalax* lipid profile and serum chemistry studies belong to the species *Spalax judaei* from the Anza population in northern Samaria Mountains [2]. All the animals were captured in the field in the same day of the experiment. Capturing the animals was performed by exposing the tunnels and trapping the animals while trying to fix the destroyed construction. The animals used for this study were female adults and ranged in weight from 80–150 g. The ethics committee of the University of Haifa approved this experiment.

#### Mouse

All Mice used in this study were C57Bl mice. The experimental protocol was approved by the Animal Care and Use Committee of the Technion.

### Humans

Blood was obtained from healthy human volunteers (n = 3), nonsmokers and under no medication. The study was approved by the Helsinki Committee of the Rambam Medical Center, Israel Ministry of Health. Participants gave a written informed consent before the blood donation.

### Blood Sampling

Blood samples were obtained from three individuals of each species (*Spalax judaei*, *Mus domesticus*, and *Homo sapiens*). For blood sampling from mole rat and mouse, animals were deeply anesthetized by intramuscular injection of Ketaset CIII (Fort Dodge, IA) at 5 mg/kg of body weight. Blood was collected directly from the heart. About 1 ml of blood was sampled from each animal, which was sacrificed immediately thereafter. Blood was pooled in separate Eppendorf tubes, centrifuged (14000 RMP, 4°C, 15 minutes), and serum collected in clean tubes. Serum was analyzed as described below. Sampling of blood from healthy human volunteers was performed through peripheral venous punctures, after 12 hours of fast.

### Serum Lipid profile determination

Serum lipid profile was assessed by commercial kits, specific for each analytes, using a diagnostic analyzer (Dimension RXL, Siemens, Germany). Total cholesterol, total triglycerides and HDL levels were measured, whereas LDL levels were calculated using the Friedewald equation. [17]

### Serum oxidation parameters

#### Plasma lipid peroxidation

Serum was incubated in the absence or presence of 100 mmol/L of the free radical generator 2, 2′-azobis-2-amidinopropane hydrochloride (AAPH; Wako Chemical Industries Ltd, Osaka, Japan) for 2 h at 37°C. AAPH is a water-soluble azo compound that thermally decomposes to produce peroxyl radicals at a constant rate. Plasma lipid peroxidation was determined by measuring the amount generated of thiobarbituric acid–reactive substances (TBARS) [26].

#### Serum paraoxonase (arylesterase activity)

Arylesterase activity was measured by using phenylacetate as the substrate. Initial rates of hydrolysis were determined spectrophotometrically at 270 nm. The assay mixture included 5 µL serum, 1.0 mmol phenylacetate/L, and 0.9 mmol CaCl_2_/L in 20 mmol tris-HCl/L, pH 8.0. Nonenzymatic hydrolysis of phenylacetate was subtracted from the total rate of hydrolysis. The extinction coefficient at 270 nm (*E*_270_) for the reaction was 1310 (mol/L)^−1^·cm^−1^. One unit of arylesterase activity is equal to 1 mmol phenylacetate hydrolyzed·min^−1^·L^−1^ [27].

### Serum chemistry

Serum samples were analyzed for general biochemical parameters, such as glucose, electrolytes (sodium, chloride), minerals (calcium, phosphor, magnesium), liver function markers (alkaline phosphatase (ALP), γ-glutamyl transpeptidase (GGT), lactate dehydrogenase (LDH)), pancreas function marker (amylase) and kidneys function markers (blood urea nitrogen (BUN), creatinine, total protein, albumin, uric acid). In addition, we have determined the serum levels of C-reactive protein, an acute phase protein released during inflammatory processes. All parameters were analyzed by commercial kits, specific for each analytes, using a diagnostic analyzer (Dimension RXL, Siemens, Germany).

### Statistical analysis

All measured serum parameters were determined from at least three different individuals of each species. Results are reported as mean±SD. Statistical analysis was performed using student T test.
